# Skin Cancers and the Contribution of Rho GTPase Signaling Networks to Their Progression

**DOI:** 10.3390/cancers13174362

**Published:** 2021-08-28

**Authors:** Alessandra Pecora, Justine Laprise, Manel Dahmene, Mélanie Laurin

**Affiliations:** 1 Oncology Division, CHU de Québec–Université Laval Research Center, Québec City, QC G1V 4G2, Canada; alessandra.pecora@crchudequebec.ulaval.ca (A.P.); justine.laprise@crchudequebec.ulaval.ca (J.L.); manel.dahmene@crchudequebec.ulaval.ca (M.D.); 2 Université Laval Cancer Research Center, Université Laval, Québec City, QC G1R 3S3, Canada; 3 Molecular Biology, Medical Biochemistry and Pathology Department, Faculty of Medicine, Université Laval, Québec City, QC G1V OA6, Canada

**Keywords:** Rho GTPase, RhoGEF, RhoGAP, skin, cancer, squamous cell carcinoma, basal cell carcinoma, melanoma

## Abstract

**Simple Summary:**

Skin cancer is the most common cancer in human. Melanoma, basal cell carcinoma and squamous cell carcinoma are the most prevalent skin cancer subtypes. A better understanding of the molecular mechanisms that contribute to the progression of skin cancer is essential due to their prevalence in the population and the emergence of resistance to current treatment for aggressive cases. The aim of our review is to provide an overview of how Rho GTPases and their regulators contribute to skin cancer progression via the perturbation of their function in the skin.

**Abstract:**

Skin cancers are the most common cancers worldwide. Among them, melanoma, basal cell carcinoma of the skin and cutaneous squamous cell carcinoma are the three major subtypes. These cancers are characterized by different genetic perturbations even though they are similarly caused by a lifelong exposure to the sun. The main oncogenic drivers of skin cancer initiation have been known for a while, yet it remains unclear what are the molecular events that mediate their oncogenic functions and that contribute to their progression. Moreover, patients with aggressive skin cancers have been known to develop resistance to currently available treatment, which is urging us to identify new therapeutic opportunities based on a better understanding of skin cancer biology. More recently, the contribution of cytoskeletal dynamics and Rho GTPase signaling networks to the progression of skin cancers has been highlighted by several studies. In this review, we underline the various perturbations in the activity and regulation of Rho GTPase network components that contribute to skin cancer development, and we explore the emerging therapeutic opportunities that are surfacing from these studies.

## 1. Introduction

Skin cancers are the most common cancers worldwide, and their incidence continues to rise [1]. The cumulative lifetime exposure to the sun is the main risk factor for developing skin cancer. Consequently, the global increase of the aging demographic combined with the improvement of skin cancer detection contribute to their increasing rate [2,3]. As a group, skin cancers are largely divided into cutaneous melanoma and non-melanoma skin cancers. Basal cell carcinoma of the skin (BCC) and cutaneous squamous cell carcinoma (cSCC) are the most frequent forms of non-melanoma skin cancers [4]. In fact, BCC is the most common cancer in humans, yet cSCC incidence is on the rise [5,6]. Melanoma, BCC and cSCC diverge in terms of their aggressiveness, cell of origin and mutational landscape [1]. Over the years, corresponding therapeutic strategies for their treatment have emerged. Nevertheless, improving our comprehension of the biology of melanoma, BCC and cSCC remains essential to improve our capacity to efficiently identify lesions with the most aggressive potentials and to develop new options for the treatment of resistant cases.

Due to their ability to regulate cytoskeletal remodeling, Rho GTPases have long been viewed as key regulators of tumor invasion [7,8]. Yet, these networks also orchestrate various cellular functions such as gene expression, cell proliferation and cell survival, that when perturbed, contribute to cancer progression [8,9]. In fact, the aberrant expression of Rho GTPases, the presence of mutations that modify their activity as well as changes in their regulation have been observed during cancer progression [8,9]. For these reasons, Rho GTPases and their regulators are interesting targets for the development of new cancer therapeutics [8,9]. Here, we discuss the current knowledge on the contribution of Rho GTPase signaling networks to skin cancer progression, and we highlight potential therapeutic opportunities.

## 2. The Normal Skin

**Figure 1:**
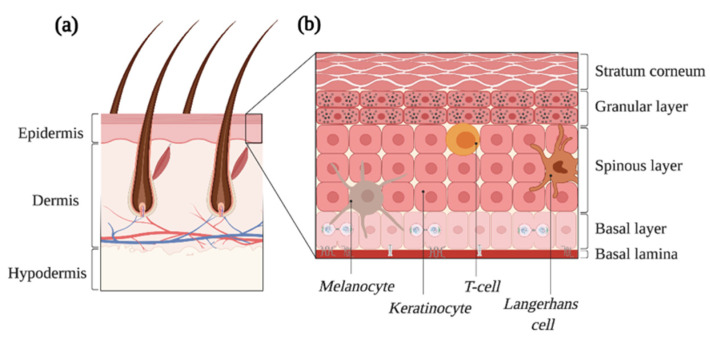
The skin is the largest organ of the human body. (**a**) The adult skin is formed of three compartments, i.e., the epidermis, the dermis and the hypodermis. Several cell types and epidermal appendages, such as the hair follicles depicted here, achieve all the skin’s essential functions. (**b**) The epidermis is a complex epithelium formed of four layers, namely the basal, the spinous, the granular and the stratum corneum as well as multiple cell types. Proliferation occurs in the basal layer, and the balance between the self-renewal and differentiation of progenitors ensures skin regeneration. Created with BioRender.com, accessed on 20 August 2021.

The skin is formed from various cell types and tissues that assemble into the body’s largest organ [10]. Amongst its manifold essential functions, the skin protects the organism by serving as a physical barrier against the external environment by preventing water loss, by participating in thermoregulation and by enabling immune surveillance [11,12,13]. The skin is formed of three main compartments, namely the epidermis, the dermis and the hypodermis (Figure 1a) [13]. In the adult, the epidermis is a complex multilayered epithelium (Figure 1b) [14]. At its base, keratinocytes, referred to as basal cells, form a monolayer that makes close contact with the basement membrane [10]. As these keratinocytes differentiate, they move outwards and transit in the spinous, granular and stratum corneum layers where dead cells are eventually shed from the skin surface [14]. The tight regulation of basal cell self-renewal, proliferation and differentiation ensures that the skin is constantly renewed throughout an individual’s lifetime [10]. Melanocytes, which are characterized by their arborized architecture, can be found in the epidermis. These neural crest-derived and melanin-producing cells are responsible for skin pigmentation, and they are key to protecting the skin against UV radiation [15]. The skin also contains immune cells that transit into the tissue to probe for the presence of intruders and barrier breaches (Figure 1b) [12].

## 3. Rho GTPases and Their Regulation

**Figure 2:**
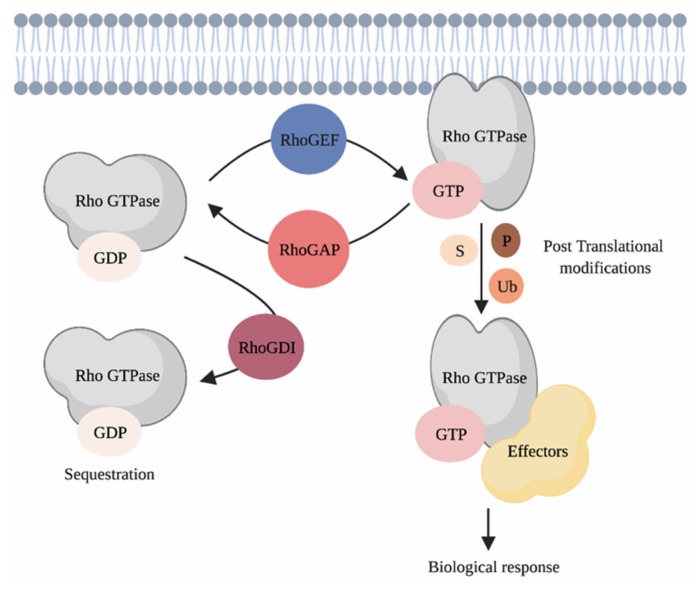
Regulating Rho GTPases. Classical Rho GTPases cycle between an inactive GDP-bound and an active GTP-bound state. This cycle is regulated by three families of proteins, i.e., the RhoGEFs, the RhoGAPs and the RhoGDIs. When bound to GTP, Rho GTPases interact with their effectors, which triggers signal transduction in cells. Post-translational modifications such as phosphorylation, ubiquitination and SUMOylation ensure the coordination of Rho GTPases activity in space and time. P = phosphorylation, S = SUMOylation, Ub = ubiquitination. Created with BioRender.com, accessed on 20 August 2021.

Rho GTPases are part of the Ras superfamily of small GTPases [16]. In humans, the 20 Rho GTPase family members are divided into eight subfamilies, i.e., the RAC, RHO, CDC42, RHOF, RHOBTB, RHOH, RHOU/RHOV and RND subfamilies that are defined based on their structural features and functions [16]. Most Rho GTPases cycle between an active guanosine triphosphate (GTP)-bound state and a guanosine diphosphate (GDP)-bound inactive conformation [17,18]. Binding of Rho GTPases to GTP triggers conformational changes that enable their binding to molecular effectors that promote signal transduction (Figure 2). This cycle is mainly synchronized by three families of proteins that account altogether for more than 150 regulators. These include the guanine nucleotide exchange factors (RhoGEFs), the guanine nucleotide activating proteins (RhoGAPs) and the guanine dissociation inhibitors (RhoGDIs) [19,20,21,22]. Rho GTPases localization, activity and stability are heavily regulated by post-translational modifications such as phosphorylation, ubiquitination, SUMOylation and lipid tail addition [23]. As we aim to focus on the role of Rho GTPase signaling networks during skin cancer progression, the interested reader is redirected to the provided references for more in depth discussions regarding the biological functions of Rho GTPases and their regulation [9,16,23,24,25,26].

## 4. Cutaneous Squamous Cell Carcinoma

### 4.1. The Pathogenesis of Squamous Cell Carcinoma

SCCs occur in several anatomical locations and are frequently found in skin, head and neck, lung and esophagus [27]. By revealing the mutational landscape of SCCs, several studies have highlighted the high degree of conservation of molecular features in SCCs from different tissues [28,29,30,31,32,33,34,35,36,37,38,39,40,41,42,43,44,45,46]. This suggests that common mechanisms are mediating the initiation and progression of these carcinomas [27]. Cutaneous SCCs result from the abnormal proliferation of keratinocytes, and they develop gradually from an actinic keratosis that progresses into invasive cSCC [47]. The mutational burden of cSCC is extremely high, and it includes mutations in *TP53*, *CDKN2A*, *NOTCH1* and *NOTCH2,* with mutations in *TP53* being the most common [29,32,38,48,49]. However, these genes are often mutated in the aged and sun-exposed skin. Therefore, other risk factors contribute to cSCC development [47,50]. These include exposure to the human papilloma virus, treatment with BRAF inhibitor, chronic wounds and environmental exposure to toxins such as arsenic [51,52,53,54,55,56,57]. An immunosuppressed microenvironment also favors cSCC progression, and transplanted patients are particularly susceptible of developing these carcinomas [58,59,60]. Cutaneous SCCs are predominantly treated via surgical resection, yet some cases are associated with high recurrence, metastasis, and death [57]. In fact, cSCCs account for about 75% of the deaths related with skin cancer, when melanoma is excluded [47]. Given the central role played by the immune component during their progression, clinical studies have emphasized the benefit of using immunotherapy for the treatment of aggressive cases [61].

In mice, the topical application of 9,10-dimethyl-1,2-benzanthracene (DMBA), which produces DNA mutations, followed by a 12-O-tetradecanoylphorbol-13-acetate (TPA) treatment that promotes hyperproliferation and inflammation, has been used extensively to recapitulate papilloma initiation and their progression to SCCs in the skin [62]. These tumors are predominantly driven by an *HRas^Q61L^* activating mutation that accounts for only a subset of human cSCCs [49,63]. A better understanding of the molecular mechanisms responsible for cSCC aggressive cases will allow us to develop tools to effectively identify the most aggressive lesions.

### 4.2. The Rho GTPases RAC1 and RHOA Act Antagonistically during SCC Progression

**Figure 3:**
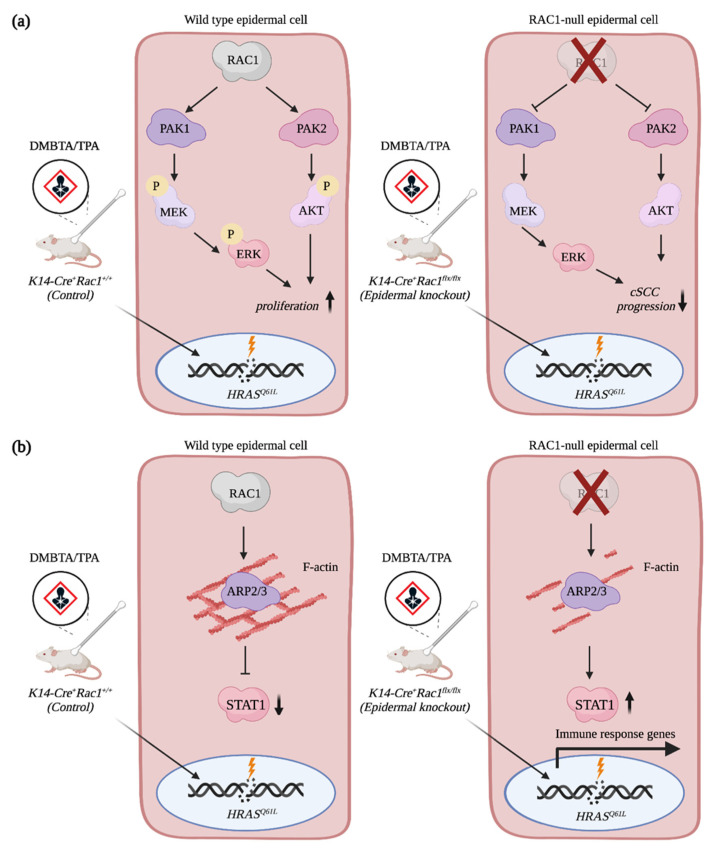
RAC1 contributes to cSCC progression. (**a**) RAC1 promotes hyperproliferation of HRAS^Q61L^-transformed epidermal cells via AKT and ERK activation. (**b**) RAC1 restricts STAT1 expression in epidermal cells by promoting F-actin assembly. Depletion of *Rac1* expression in keratinocytes increases levels of STAT1 and immune response genes, which hypersensitizes keratinocytes to inflammatory stimuli. Created with BioRender.com, accessed on 20 August 2021.

RAC1 regulates multiple signaling pathways during SCC progression. High levels of RAC1 activity and mutations in the *RAC1* gene have been observed in SCCs [38,64]. In mice, studies have shown that the specific deletion of *Rac1* in keratinocytes is sufficient to reduce the tumor burden of animals treated with DMBA/TPA when compared to treated controls (Figure 3a) [65]. Treatment of mice with the NSC23766 compound, which inhibits RAC signaling, also lessens the number and volume of tumors induced by DMBA/TPA treatment [66]. Mechanistically, RAC1 mediates hyperproliferation of RAS transformed keratinocytes by promoting ERK and AKT activation via the phosphorylation of MEK and PAK2, respectively (Figure 3a) [65]. Intriguingly, RAC1 also orchestrates the signaling crosstalk between the keratinocytes and the immune cells. By promoting the formation of filamentous actin (F-actin), RAC1 restricts STAT1 expression. In *Rac1-null* keratinocytes, F-actin levels are reduced, which promotes expression of STAT1 and immune response genes in the epidermis (Figure 3b) [67]. Altogether, this hypersensitizes the *Rac1*-null epidermis to inflammatory stimuli [67]. It will be interesting to further dissect the contribution of RAC1 given the fundamental role played by the immune system during SCC progression [68,69]. Remarkably, expression of RAC^V12^ in the epidermis of mice recapitulates hallmarks of human psoriasis, which includes important immune infiltration. Altogether, this highlights the involvement of RAC1 in modulating the immune response in the skin [68,69].

SCC formation can also be modelled in mice via their exposition to UVB radiation that triggers a DNA damage response. Intriguingly, this response is attenuated in the *Rac1*-null epidermis, which likely facilitates SCC development [70]. Hence, depending on the mutational signature of epidermal cells, RAC1-signaling either favors or prevents cSCC progression. Notably, mice that are defective in TGFβ signaling develop aggressive SCCs that have high RAC-mediated signaling [71]. These tumors also upregulate the expression of several Rho GTPase network components such as *Rac2*, *Rhoh*, *Rhoj*, *Vav1*, *Dock2* and *Elmo1* [71]. Apart from these processes, RAC1 contributes to the engulfment of apoptotic SCC cancer cells by epithelial nonprofessional phagocytes [72]. Inhibition of RAC1 also improves the sensitivity of head and neck SCCs to ionizing radiation and cisplatin treatment [73,74]. Whether these mechanisms are conserved in SCCs from the skin remains to be determined, yet these studies underscore the relevance of tightly regulating RAC1 activity to maintain epidermal homeostasis.

**Figure 4:**
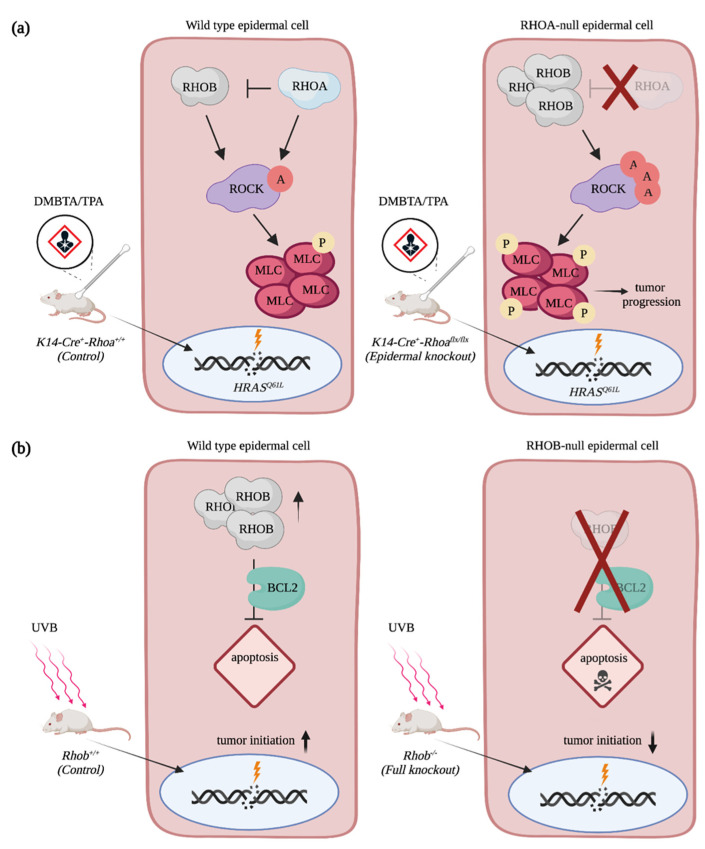
RHOA and RHOB contribution to cSCC progression. (**a**) RHOA impedes cSCC progression by restricting levels of RHOB in HRAS^Q61L^-transformed keratinocytes. In the RHOA-null epidermal cells, RHOB levels are increased, which promotes RHO-mediated signaling. (**b**) RHOB protects cells from UVB-induced apoptosis via BCL2, which enables cutaneous tumor formation. In the RHOB-null epidermal cells, UVB exposition promotes cell death. A = activation, P = phosphorylation. Created with BioRender.com, accessed on 20 August 2021.

Members of the RHOA subfamily also contribute to cSCC progression. Mice with a skin specific deletion of *Rhoa* have a higher tumor burden and develop more aggressive tumors than control animals upon DMBA/TPA treatments [75]. This suggests that RAC1 and RHOA play antagonistic roles during skin carcinoma progression, as is often observed in other cancers [9]. Intriguingly, an increase in RHO-mediated signaling characterized by ROCK activation and phosphorylation of myosin light chain is observed in the *Rhoa*-depleted epidermis due to a rise in RHOB protein levels (Figure 4a). In fact, RHOB expression is required to promote hyperproliferation and invasion of RHOA-null keratinocytes. In the transformed epidermal cells, depletion of RHOA prevents lysosomal degradation of RHOB, while also increasing the fraction of RHOB at the cell membrane (Figure 4a). Whether RHOB acts as a tumor suppressor or as an oncogene on its own remains unclear. Contrary to what one might have expected, *Rhob* knockout mice are more sensitive to DMBA/TPA treatment, and they develop more papilloma than the treated control mice [76]. However, the depletion of RHOB in the other tissues, such as in the immune cells, might contribute to the increase in the number of tumors. A skin-specific deletion of *Rhob* will be required to address this. Moreover, it is possible that RHOA contributes to the observed phenotype, which was not addressed [76]. The mutational signature of skin tumors also alters RHOB-mediated signaling. In fact, *Rhob* knockout mice develop fewer papilloma than wild type animals when exposed to UVB. RHOB expression is induced in keratinocytes following ultraviolet treatment, and this contributes to the protection of cells from apoptosis both in vitro and in vivo via a BCL2-mediated mechanism (Figure 4b) [77,78]. In the absence of RHOB, an increase in cell death is observed, and it results in the formation of fewer lesions on the skin of mice. Still, the tumors that developed in the *Rhob* knockout animals are less differentiated and more aggressive than the controls, which implies that RHOB has multiple roles during cSCC progression [77].

Studies using SCC cells from other tissues have also underscored the contribution of RHO-mediated signaling. The downregulation of miR-138 promotes RHOC and ROCK2 expression, while the downregulation of miR-340 correlates with an increase in RHOA expression [79,80]. Moreover, Abraham et al. identified RHOA as a negative regulator of proliferation using a CRSIPR-mediated screen in lung SCC cells that overexpress ΔNp63α, which is associated with a poor prognosis for patients [81]. Mutations in the effector binding domain of RHOA were also identified in head and neck SCCs, and the majority of these tumors show a downregulation of RHOA mRNA [75,82]. Altogether, this reinforces the idea that regulation of RHOA activity is central for SCC progression [83].

### 4.3. The Contribution of Rho GTPases Regulators to SCC Progression

Given the plethora of roles played by RAC1, it is not surprising to observe the expression of numerous RhoGEFs such as *TIAM1*, *VAV2* and *VAV3* in cutaneous papilloma and SCCs [84]. Much like *Rac1*-epidermal null animals, *Tiam1* knockout mice are more resistant than control animals to DMBA/TPA treatment [85]. However, TIAM1-null tumors that do develop are of higher grade, which indicates that this RhoGEF plays multiple and antagonistic functions during cSCC development [85]. Intriguingly, TIAM1 interacts with active RAS to promote PI3K-mediated activation of RAC [86]. Whether this mechanism is at play in cSCC tumors remains to be determined. When subjected to DMBA/TPA treatment, the *Vav2^−/−^Vav3^−/−^* double knockout animals develop tumors with a slower kinetic than control mice, and much like the *Rac1*-epidermal null animals, their tumor burden is reduced [84]. *Vav2^−/−^Vav3^−/−^* keratinocytes were shown to be more prone to cell death than control keratinocytes following DMBA/TPA treatment. This likely prevents the accumulation of detrimental mutations in the double mutants. Mechanistically, VAV2 and VAV3 act downstream of cPKC and FYN to promote RAC, ERK and STAT3 activation to favor cell cycle entry [84]. These RhoGEFs also foster the expression of extracellular factors such as HGF and FGF7 and create an autocrine/paracrine mechanism responsible for cell survival, hyperproliferation and the formation of a proinflammatory environment [84]. Mice that express a catalytically hypomorphic VAV2 are also partially impaired in their ability to develop tumors following DMBA/TPA treatments [87]. In contrast, expression of a constitutive active form of the RhoGEF in the epidermis triggers severe hyperplasia, which is associated with a stem-like phenotype that is c-MYC- and YAP-dependent [88]. In human, *VAV2* mRNA has been shown to increase progressively in cSCC, and high mRNA levels correlate with poor prognosis in HPV head and neck SCC [88]. Altogether, this suggests that targeting VAV2 would be beneficial to prevent the initiation and progression of cSCC.

Few studies have addressed the role of other Rho GTPase family members. *RND3* mRNA and protein are downregulated in esophageal SCC tissues and cell lines. Moreover, RND3 overexpression inhibits cell growth, which suggests that it could act as a tumor suppressor [89,90]. Still a complete picture of the contribution of Rho GTPase signaling network components during cSCC progression is greatly lacking. Given the high number of Rho GTPases and their regulators, unbiased screening approaches would be beneficial to address this. Ultimately, the development of cSCC mouse models that are not driven by oncogenic HRAS will also be instrumental to delineate the full role of Rho GTPase signaling networks [29].

## 5. Basal Cell Carcinoma of the Skin, the Most Common Cancer Worldwide

### 5.1. Basal Cell Carcinoma Is Triggered by Hedgehog Signaling

BCC is the most frequent cancer in human, and it accounts for 50% of all cancers in the United States [1]. BCCs are prevalent in the Caucasian aging population as they mainly result from a lifetime exposure to the sun [91,92]. Surgical resection is the main treatment for this cancer. While the mortality rate associated with BCC is negligible, this cancer can considerably impact the life of patients by causing important skin destruction. Additionally, disease recurrence is commonly observed and can be linked with substantial morbidity [93]. Critically, BCC inflicts a significant financial burden to the health care system due to the tremendous number of cases.

**Figure 5:**
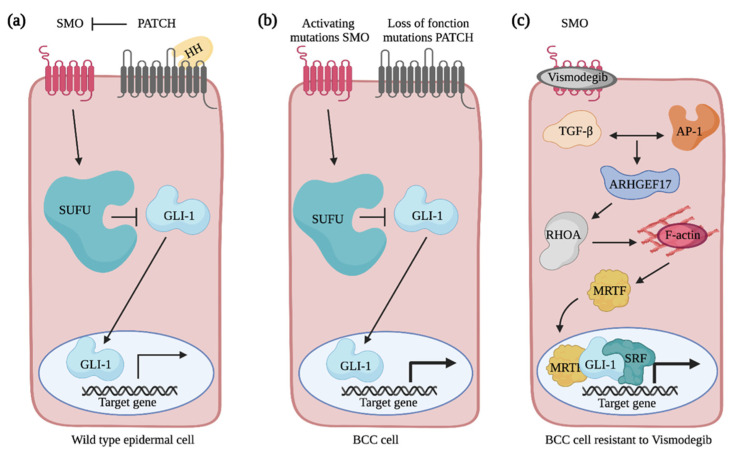
Overview of Hedgehog signaling pathway in normal and basal cell carcinoma cells. (**a**) The Hedgehog signaling pathway is activated when Hedgehog binds the PATCHED (PATCH) receptor. This relieves the inhibition on SMO by PATCH. By sending signals through a series of interacting proteins, including suppressor of fused (SUFU), SMO activates the downstream GLI family of transcription factors. (**b**) In BCC cells, SMO activating mutations or PATCH loss of function mutations amplify GLI transcriptional activity and promote the constitutive activation of the Hedgehog signaling pathway. (**c**) Non-canonical activation of GLI-mediated transcription in vismodegib-resistant basal cell carcinoma cell. TGFβ and AP-1 signaling pathways promote ARHGEF17 expression that activates RHOA. By promoting actin polymerization, RHOA induces the translocation of MRTF in the nucleus and the formation of the MRTF-SRF complex that acts as a transcriptional cofactor for GLI to regulate a subset of target genes and to mediate resistance of the BCC cells to treatment. Created with BioRender.com, accessed on 20 August 2021.

Mutations leading to the constitutive activation of the Hedgehog signaling pathway in epidermal cells are the hallmark of BCC pathogenesis [94]. In mammalian cell, Hedgehog signaling is achieved when one of the three Hedgehog extracellular ligands, i.e., Sonic Hedgehog, Indian Hedgehog and Desert Hedgehog, binds the PATCHED receptor (Figure 5a). Binding of ligand releases the inhibition that PATCHED maintains on the transmembrane protein Smoothened (SMO), which culminates with the activation of the GLI transcription factors that are usually sequestered in the cytoplasm by other proteins such as the Suppressor of Fused (SUFU). In BCCs, PATCHED loss-of-function or SMO activating mutations amplify GLI transcriptional activity and trigger hyperactivation of Hedgehog signaling in epidermal cells (Figure 5b) [94]. To this date, advanced and/or metastatic BCCs are treated with the SMO inhibitors vismodegib and sonidegib [95,96,97]. Unfortunately, this treatment succeeds in only 50% of patients, of which 20% will develop resistance [95,98,99,100,101]. Additionally, studies have highlighted that in half of the vismodegib-resistant patients, additional mutations in Hedgehog signaling components are neither acquired nor responsible for the newly developed resistance [102]. This strongly suggests that alternative signaling pathways might be involved in disease progression. Due to the high risk of skin destruction associated with BCCs, their frequent occurrence and the resistance arising from current therapies, it is crucial to seek novel treatment strategies based on a better understanding of BCC biology.

### 5.2. Rho GTPases and the Non-Canonical Activation of GLI Transcriptional Activity

Upon hyperactivation of the Hedgehog signaling pathway in epidermal cells, drastic cytoskeletal rearrangements are observed that ultimately drive tumor cells invasion into the skin’s deep layers [103,104]. In the transformed cells, genes encoding for extracellular matrix and cytoskeletal components are among the ones showing the most increase in their expression [104,105]. Rho GTPase signaling networks are good candidates to mediate the cytoskeletal rearrangements associated with BCC progression. Nevertheless, their contribution has remained poorly addressed. While CDC42 expression is increased in BCC tumors when compared to the normal epidermis, its contribution to BCC pathogenesis has yet to be determined [106]. In addition to mutations in the canonical components of the Hedgehog signaling pathway, it is becoming clear that the regulation of GLI transcriptional activity via non-canonical mechanisms contributes to the survival of SMO inhibitor-resistant cells [95,107]. Particularly, cytoskeletal regulation by RHOA was shown to regulate GLI1 activity in a non-canonical manner and to confer resistance to vismodegib treatment in a BCC mouse model [108]. In these cells, active RHOA promotes actin polymerization and translocation of the MRTF transcriptional activator into the nucleus (Figure 5c) [109]. This favors formation of the SRF–MRTF complex that acts as a transcriptional cofactor for GLI1 [108]. Altogether, this reinforces the expression of a subset of Hedgehog target genes [108]. Cooperation between TGFβ and AP-1 signaling is required to activate RHOA via the transcriptional regulation of several RhoGEFs, such as *ARHGEF17* [110]. In the future, it will be interesting to further investigate the role played by these RhoGEFs and to test whether they play distinct or redundant functions to facilitate the emergence of resistant cells.

BCC initiation mimics the cellular events associated with hair follicle morphogenesis [103]. During embryonic development, unspecified epidermal progenitors that accumulate sufficient WNT instructive cues via epithelial and mesenchymal signaling crosstalk initiate hair follicle development by forming hair placode [111,112,113,114]. Once specified, placodes invaginate into the dermis, a progression that requires Sonic Hedgehog and cytoskeletal remodeling [115,116,117,118,119,120]. Hair follicle development is completed via the differentiation of cells into the hair lineages [121]. In adults, epidermal cells that activate oncogenic Hedgehog signaling reprogram their fate to adopt a gene expression profile that shares high similarities with embryonic hair follicle cells [103,107]. These similarities, coupled with the involvement of Hedgehog signaling in both processes, provide compelling support for the hypothesis that the identification of new targets for BCC treatments should focus on the downstream developmental regulators that fuel hair follicle downgrowth. We recently developed a unique screening approach to identify regulators of hair follicle morphogenesis [122]. Using this strategy, we functionally tested the myriad of Rho GTPases, RhoGEFs, RhoGAPs and RhoGDIs (150 genes) for their involvement during hair follicle formation and successfully identified new regulators of hair follicle invasion among them [122]. In the future, it will be interesting to test the contribution of these candidates during Hedgehog mediated epidermal proliferation and invasion, as these are likely to be reactivated during BCC progression.

## 6. Melanoma, a Highly Invasive Cancer

### 6.1. Melanoma, the More Aggressive Skin Cancer

Melanoma is a heterogenous disease that originates from the pigment-generating cells, the melanocytes [123]. Cutaneous melanoma results from the oncogenic transformation of melanocytes located in the epidermal layers [123,124]. Exposure to UV, family history and fair skin are among the major risk factors for developing melanoma [125]. Even if melanoma accounts for only 5% of skin cancers, it is responsible for more than 60% of the deaths attributed to skin cancers [123]. The high propensity of melanoma cells to invade the surrounding tissue and to metastasize, even at very early stages, is at the root of their poor prognostic. Several genetic alterations are responsible for melanoma development, and these often correlate with anatomical location and histological characteristics. The most frequent perturbations associated with cutaneous melanoma are activating mutations in *BRAF* and *NRAS* as well as inactivating mutations in *NF1* [126,127]. Substitution of valine 600 for a glutamic acid (BRAF^V600E^) is the most common BRAF mutation [53,128]. Expression of this variant results in the constitutive activation of the MAPK kinase pathway in melanocytes [129]. Still, the *BRAF^V600E^* mutation is observed in benign lesions, which suggests that additional signaling pathways contribute to melanoma progression [130]. Melanoma can be excised from the skin when caught early. However, systemic treatments are required when metastases are present. These treatments usually involve a combination of BRAF and MEK inhibitors [131,132]. Vemurafenib, a drug that specifically targets mutated BRAF^V600E^, received FDA approval in 2011 [133]. However, not all melanoma patients carry the BRAF^V600E^ activating mutation, and a significant portion of patients are either initially resistant to the drug or they develop resistance to this treatment. Other more recent and promising options include immune checkpoint blockage therapies [131,134,135,136,137]. Yet again, high intratumor heterogeneity has been suggested to be an important factor towards the development of treatment resistance. Hence, there is a constant need for a better understanding of the molecular mechanisms responsible for melanoma progression.

### 6.2. The Discovery of a Fast-Cycling Rho GTPase

**Figure 6:**
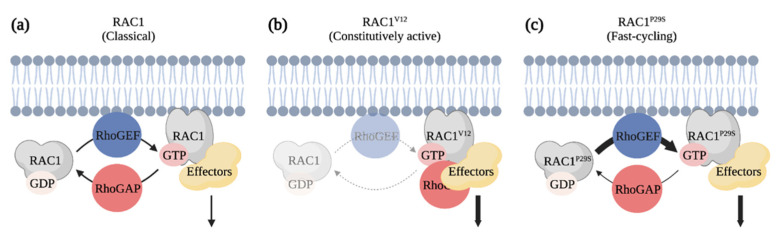
RAC1^P29S^ is a fast-cycling Rho GTPase identified as an oncogenic driver of melanoma. (**a**) Classical Rho GTPases cycle between GDP-bound inactive and GTP-bound active forms. (**b**) RAC1^V12^ is defective in its GTPase activity and constitutively bound to GTP. Expression of this mutant has the potential of titrating RhoGAPs and effectors from other signaling pathways in cells. (**c**) RAC1^P29S^ is a fast-cycling Rho GTPase. The substitution of amino acid in the Switch I domain leads to a gain of function that enhances RAC1-mediated signals. Created with BioRender.com, accessed on 20 August 2021.

Melanoma cells have the ability to adjust quickly to their environment and to switch between a mesenchymal and amoeboid migration mode, which likely contributes to their aggressiveness and poor prognosis [138,139,140,141]. Given that this balance is regulated by levels of active RAC and RHOA, it is not surprising that these two Rho GTPases are involved during melanoma progression [138]. Notably, whole genome sequencing experiments have revealed that RAC1 is frequently mutated in melanoma patients [142,143]. In fact, up to 9.2% of sun-exposed melanoma contain a *RAC1^P29S^* mutation that replaces the proline 29 for a serine in the switch I domain of RAC1 that is responsible for nucleotide and effector binding [143]. Unlike mutations observed in RAS that maintain the GTPase in a constitutively GTP-bound active form, the P29S mutation in RAC1 creates a fast-cycling GTPase that enhances RAC1-mediated signaling in cells (Figure 6) [144]. Melanoma cells expressing *RAC1^P29S^* are particularly resistant to RAF and MEK inhibitors, while being sensitive to PAK inhibitors [145,146]. This ability of RAC1 to cycle between a GDP-bound and GTP-bound form seems to be fundamental to its oncogenic role, since expression of RAC^V12^, which is constitutively bound to GTP, is unable to initiate melanocyte neoplasia on its own in the zebrafish [147,148]. Genetic depletion of *Rac1* in mice or treatment with a RAC inhibitor prevents the growth of melanocyte tumors when *NRAS^Q61K^* expressing melanocytes are grafted into mice [148]. The requirement of RAC-mediated signaling is also highlighted by changes in the activity of its upstream regulators. Mutations in the RhoGEF *PREX2* were identified in melanoma patients, and expression of mutated PREX2 proteins in melanoma cells accelerates tumor growth following their grafting in mice [149] Moreover, deletion of *Prex1* expression prevents melanocyte migration during mice development [150]. Additionally, deletion of *Prex1* in the *Tyr:Nras^Q61K^;INK4a^−/−^* melanoma mouse model results in the development of less metastasis to the lung than controls [150]. The RhoGEF TIAM is also overexpressed in a cohort of melanoma patients, and it was suggested that TIAM1 regulates RAC to modulate CADHERIN expression during melanoma cell invasion [147,151]. Altogether this suggests that targeting RAC1-mediated signaling could be of value in specific melanoma cases.

In contrast to *RAC1*, no mutations in *RHOA* have been detected thus far in melanoma; still, low expression of *RHOA* was reported to be associated with a poorer prognostic in a cohort of melanoma patients [152]. To date, numerous in vitro studies have investigated the contribution of RHOA-mediated signaling in melanoma cells [153,154,155,156,157,158,159,160]. Notably, it was shown that expression of the dominant negative form, RHOA^N19^, sensitizes melanoma cells to UV radiation [153]. Another study highlighted that FASL expression is regulated by RHOA, which could be important for melanoma cells to escape immune surveillance [156]. Whether these mechanisms are at play in vivo has yet to be tested. The analysis of RHOA contribution using cell line xenografts has led to conflicting conclusions. Inhibition of the RHOA effector ROCK via the treatment of mice with the Y-27632 compound impairs the growth and invasion of B16 melanoma cells following their transplantation [161]. Similar results were observed when ROCK was inhibited using the AT13148 and CCT129254 compounds [162,163,164]. Altogether, this hints towards RHO mediating melanoma progression. However, treatment of mice with Y-27632 following the injection of melanoma cells harboring a BRAF mutation promotes tumor growth [165]. This suggests that the mutational signature of melanoma cells determines whether RHOA acts as an oncogene or a tumor suppressor. In the future, the use of genetic mouse models will be extremely useful to test this hypothesis.

The contribution of other Rho GTPase network components to melanoma progression is slowly emerging. Notably, a *CDC42^G12D^* mutation was identified in patients, yet its role remains to be tested [142]. In vitro studies have revealed that CDC42 is required downstream of the RhoGEF DOCK10 to favor amoeboid migration in melanoma cells [141]. Moreover, CDC42 contributes to the release of exosomes when malignant melanoma cells are treated with WNT5A [166]. Among other Rho GTPases, RHOJ is overexpressed in metastatic melanoma, and it was shown to mediate the chemoresistance of these cells by suppressing mechanisms that sense DNA damage and by promoting invasion [167,168,169]. Importantly, deletion of *Rhoj* in a mouse model of melanoma driven by the expression of *BRAF^V600E^* and by the deletion of *PTEN* reduces tumor onset [168]. Fewer metastases were observed in the lung of *Rhoj* mutants when compared to the control animals [168]. Mechanistically, expression of RHOJ in melanoma promotes PAK activation, which favors BAD phosphorylation and cell survival [168]. In fact, transcriptomic analysis revealed that the expression of metabolic and pro-apoptotic genes is higher in the RHOJ-null melanoma when compared to the control tumors. Interestingly, treatment of mice with the FRAX597 PAK inhibitor inhibits melanoma growth in mice [167]. Altogether, this suggests that targeting the RHOJ–PAK axis would be beneficial for melanoma patients with BRAF mutations.

## 7. Conclusions

Over the years, the worldwide population has become increasingly aware of the risk associated with prolonged sun exposure, yet with the increase of the elderly population and the development of better detection tools, we are still observing a rise in the incidence of skin cancers. Melanoma, cSCCs and BCCs are the more prevalent skin cancers. While key mutations have been associated with their pathogenesis, a better understanding of the biology of these cancers is required to quickly identify the lesions with the most aggressive potential. Rho GTPase signaling networks have long been recognized as important regulators of cancer invasion. Nevertheless, it is now becoming clear that they could also be directly involved during tumor development, while also contributing to tumor resistance. Recent sequencing efforts have also revealed a new class of fast-cycling GTPases. Still, given the number of Rho GTPases and their regulators, high-throughput genetic models will likely be beneficial to probe the role of these proteins in the transformed skin. Ultimately, this could improve our detection of lesions with the most aggressive potential.

## Data Availability

The data presented in this study are available on request from the corresponding author.
